# Trigger-day progesterone-to-mature-oocyte ratio, cycle-level blastocyst yield, and recorded embryo-transfer outcomes: a retrospective cohort study

**DOI:** 10.3389/fendo.2026.1938120

**Published:** 2026-10-28

**Authors:** Yidong Wei, Yu Xu, Nuanfang Wei, Yu Chen, Yupei Zhang, Xueli Li, Haifan Wei

**Affiliations:** 1 Department of Clinical Laboratory, Liuzhou Hospital, Guangzhou Women and Children’s Medical Center, Liuzhou, China; 2 Department of Clinical Laboratory, Guigang Clinical Medical Research Center for Medical Laboratory, Guigang City People’s Hospital, Guigang, China; 3 Liuzhou Municipal Liutie Central Hospital, Liuzhou, China

**Keywords:** blastocyst yield, embryo transfer, IVF/ICSI, live birth, P/MII ratio, progesterone

## Abstract

**Background:**

The progesterone-to-metaphase II oocyte ratio (P/MII) combines trigger-day progesterone with mature oocyte count after retrieval. Its associations may reflect ovarian response and embryo allocation.

**Methods:**

This retrospective cohort included 7,778 *in vitro* fertilization/intracytoplasmic sperm injection (IVF/ICSI) cycle records. Existing pregnancy analyses used 4,273 positive-transfer records. A linked clinical and laboratory source export enabled *post hoc* analyses using recorded extended culture counts, retrieval order, transfer dates, thawing records, and source-identifier grouping.

**Results:**

The original high-versus-low P/MII rate ratio for blastocysts per recorded 2PN zygote was 0.796 (95% CI 0.762–0.831). Among the same 5,821 culture-positive records, the adjusted rate ratio was 0.934 (0.902–0.968) with a 2PN denominator and 0.974 (0.945–1.005; P = 0.101) with the recorded culture denominator. The continuous culture-count association remained small (aRR 0.993 per 0.01, 0.989–0.997). The source recorded 6,148 first and 1,630 later retrievals. In 3,526 first-retrieval, date-consistent records without thawing evidence, the ratio term after inclusion of progesterone and MII was associated with clinical pregnancy flags (aOR 0.952, 0.919–0.986; P = 0.006) but was uncertain for live birth flags (0.968, 0.935–1.002; P = 0.067). Source-identifier-grouped live birth areas under the receiver operating characteristic curve (AUCs) were 0.623 with the components, 0.623 with P/MII, and 0.624 with all three.

**Conclusions:**

Recorded associations depended on the culture population, denominator, and outcome. Incremental clinical usefulness was not established. These data do not support a physiological ratio mechanism or a clinical cutoff for trigger-day or freeze-all decisions.

## Introduction

Controlled ovarian stimulation in *in vitro* fertilization (IVF) and intracytoplasmic sperm injection (ICSI) is intended to generate a cohort of mature oocytes, but it also produces supraphysiological steroid exposure. Circulating progesterone may therefore rise during the late follicular phase before final oocyte maturation triggering (1–3). Whether this elevation compromises outcomes after fresh embryo transfer remains debated. Early studies reported no clear relationship between trigger-day progesterone and pregnancy (4), whereas large cohorts and a meta-analysis of more than 60,000 cycles associated higher concentrations with a lower probability of pregnancy after fresh transfer (5–7). Subsequent evidence suggests a more complex pattern: both low and high concentrations may be associated with lower live birth rates (7, 8), the magnitude of the association may vary with ovarian response (9), and findings in women with polycystic ovary syndrome (PCOS) have been inconsistent (10–12). Premature advancement of endometrial maturation and resulting embryo–endometrium asynchrony are among the leading proposed mechanisms (13, 14).

Previous studies have divided progesterone concentration by follicle number, retrieved oocyte number, or MII oocyte number to construct indices such as the progesterone-to-follicle index and progesterone-to-metaphase II oocyte ratio (P/MII) (15–20). These calculations describe progesterone relative to a measure of ovarian response; they do not establish a physiological measure of endometrial progesterone exposure or receptivity. In particular, P/MII is a deterministic function of progesterone concentration and mature oocyte count, and its denominator is itself associated with IVF outcomes. Associations with clinical pregnancy or live birth have been reported in selected IVF/ICSI populations (17, 19, 20), but an association of the ratio with an outcome does not demonstrate additional information beyond its components or an independent hormonal effect.

We examined IVF/ICSI cycle records in a single-center retrospective cohort to evaluate whether P/MII was associated with recorded cycle-level blastocyst yield per recorded 2PN zygote,good-quality Day-3 embryo yield, and recorded pregnancy outcomes among positive-transfer records. We also assessed whether the ratio added information beyond progesterone concentration and MII count considered separately and jointly. An internally derived changepoint and comparisons across database-recorded ovarian response labels were exploratory analyses. The study was designed to estimate associations in recorded treatment data, not to establish a biological mechanism, a culture-specific blastulation rate, a causal effect of altering the ratio, or a clinically actionable threshold.

## Materials and methods

### Study design and participants

We assembled a single-center retrospective cohort of recorded treatment cycles for women who underwent assisted reproductive technology (ART) at the Reproductive Medicine Center of Liuzhou Hospital, Guangzhou Women and Children’s Medical Center, between 1 August 2020 and 31 December 2024. An expanded clinical and laboratory source export contained 15,436 unique assisted reproductive technology (ART) cycle keys and linked one-to-one to all 7,778 analytic records. The linked records contained 6,647 distinct values of a source identifier labelled total number; 913 identifier groups contained multiple analytic cycles. The source identifier was used solely as an observed grouping label, rather than as a verified patient key. The study is reported in accordance with the Strengthening the Reporting of Observational Studies in Epidemiology (STROBE) statement (21).

The analytic dataset comprised 7,778 cycles, with recorded ages of 20–40 years. The source retrieval-order field identified 6,148 first and 1,630 later retrievals; the full cohort was therefore not restricted to first-cycle records. Laboratory outcomes were evaluated in this selected retrieval cohort. Existing pregnancy analyses included 4,273 records with a positive embryo transfer count; the remaining 3,505 had no recorded transfer and were not all elective freeze-all cycles. A separate 21,526-key registry linked all analytic records but did not reconstruct the upstream eligibility and exclusion sequence. The expanded export supplied retrieval and transfer dates: all 4,273 positive-transfer intervals were 3, 5, or 6 days. Fifteen of these records also had positive thaw counts and valid thaw identifiers, leaving individual embryo origins uncertain. *Post hoc* analyses therefore separately examined date-consistent records with no thawing evidence and those additionally recorded as first retrievals. Neither date consistency nor absence of thawing records establishes the origin of every transferred embryo.

### Ovarian stimulation, triggering, and luteal support

Baseline evaluation included age, infertility history, body mass index (BMI), transvaginal-ultrasound antral follicle count (AFC), anti-Müllerian hormone (AMH), and serum follicle-stimulating hormone (FSH), luteinizing hormone (LH), estradiol (E2), and progesterone on cycle Day 2 or 3. The starting gonadotropin dose was individualized according to age, ovarian reserve, and anticipated response and was subsequently adjusted using serial ultrasonography and serum E2. Recorded agonist regimens included long, ultralong, and short protocols; antagonist regimens were recorded separately. All 527 progestin-primed controlled ovarian stimulation (PPCOS)-coded records mapped to the source description stimulation under high progestogen levels. The Others category comprised mild stimulation (183 records), luteal-phase stimulation (44), and natural cycles (3). We retained this source category; medication-level details were unavailable for classifying a standardized progestin-primed ovarian stimulation (PPOS) regimen.

Final oocyte maturation was triggered with human chorionic gonadotropin (hCG), a GnRH agonist or a dual trigger, according to ovarian reserve, response and the estimated risk of ovarian hyperstimulation syndrome. Oocytes were retrieved 34–36 h later by transvaginal ultrasound-guided aspiration. Conventional IVF or ICSI was selected according to semen parameters and the infertility diagnosis. Normal fertilization was assessed 16–18 h after insemination by the presence of two pronuclei (2PN). When fresh transfer was considered, endometrial thickness was monitored and transfer proceeded if trigger-day endometrial thickness was ≥7 mm and no clinical contraindication was present. Luteal support with vaginal, oral, and/or intramuscular progesterone began on the day of oocyte retrieval and continued to approximately 10 weeks of gestation when pregnancy was confirmed.

### Exposure

P/MII was calculated as the serum progesterone concentration measured on the trigger day (ng/ml) divided by the number of MII oocytes recorded after retrieval. The ratio became available only after retrieval and maturity assessment. It was a derived statistical measure, not a post-retrieval progesterone measurement. MII ascertainment depended on fertilization method. In ICSI cycles, maturity was confirmed directly after oocyte denudation under microscopy. Conventional IVF cycles used the laboratory-recorded number of mature oocytes. The linked registry provided the fertilization method for every analytic cycle: 5,450 conventional IVF cycles, 2,327 ICSI-based cycles, and one mixed IVF+ICSI cycle. The ICSI-based group comprised 1,704 ICSI, 615 rescue ICSI, and eight late ICSI cycles. Existing method-stratified sensitivity analyses compared conventional IVF with the ICSI-based group and excluded the single mixed cycle.

### Hormone measurements

FSH, LH, E2, and progesterone were measured at baseline and on the trigger day using a chemiluminescent microparticle immunoassay on an ARCHITECT i2000SR analyzer (Abbott Laboratories, Abbott Park, IL, United States). The laboratory information system recorded progesterone values below the reporting limit as “<0.10 ng/ml.” In the de-identified analytic export available for modeling, 135 trigger-day progesterone records appeared at 0.10 ng/ml, and none were lower. We therefore treated these observations as left-censored boundary records rather than exact measured concentrations. Sensitivity analyses excluded boundary records and separately replaced 0.10 with 0.01, 0.05, or 0.099 ng/ml. Serum beta-hCG was measured 12–14 days after embryo transfer.

### Outcomes

The embryological outcomes were recorded blastocyst count and good-quality Day-3 embryo count. In the original analysis of all 7,778 records, blastocyst count divided by recorded 2PN count defined cycle-level recorded blastocyst yield per recorded 2PN zygote. This denominator included embryos allocated to cleavage-stage transfer or cryopreservation and to extended culture. In 173 records, blastocyst count exceeded 2PN count, preventing interpretation as a proportion of 2PN-derived embryos forming blastocysts. The expanded source supplied a recorded extended culture count for every analytic cycle; 5,821 had a positive count, and blastocyst count never exceeded that count. We used the recorded culture count as an alternative denominator in *post hoc* sensitivity analyses. Its internal consistency with the source available blastocyst rate did not establish the pronuclear origin or retrieval source of each cultured embryo. The endpoint is therefore recorded blastocyst yield per recorded cultured embryo, not a verified 2PN-specific biological blastulation rate. Good-quality Day-3 yield retained the recorded 2PN denominator. Pregnancy endpoints were the existing implantation, clinical pregnancy, miscarriage, and live birth record flags in the selected transfer cohort.

Pregnancy analyses consumed existing binary record flags. Implantation positivity corresponded exactly to a positive source implantation count. The stated clinical pregnancy definition required an intrauterine gestational sac with fetal cardiac activity, but the expanded export did not fully substantiate those ultrasound criteria for every positive flag. The miscarriage flag mapped exactly to the recorded loss category; 149 of 331 positive flags lacked a loss week and two had a recorded week of at least 24, so the intended loss-before-20-weeks definition could not be independently validated. Live birth flags mapped to recorded term, preterm, or delivered categories; 2,015 of 2,016 had positive live infant counts, and 749 lacked delivery gestational age. Delivery beyond 24 completed weeks was therefore not verified for every flagged record. Existing flags were preserved for reproducibility rather than clinically re-adjudicated or silently replaced.

### Ovarian response phenotypes

Ovarian response labels were assigned as mutually exclusive clinical database categories at consultation: normal ovarian response (NOR), PCOS, or poor ovarian response (POR). The phenotype definitions followed a published three-category scheme (22). PCOS was defined according to the revised Rotterdam consensus, requiring at least two of oligo- or anovulation, hyperandrogenism, and polycystic ovarian morphology (23). POR was assigned for diminished or poor ovarian response features, including AFC <5, AMH <1.1 ng/ml, or a documented prior poor ovarian response when present in the clinical history. AFC and AMH were available in the analytic export, but the original phenotype classification was not independently reconstructed from all diagnostic source components. Women with regular menstrual cycles and normal ovarian reserve who met neither PCOS nor POR criteria were classified as NOR. The classification priority was PCOS first, then POR, then NOR. Phenotype analyses were exploratory, NOR was the reference category, and effect modification was evaluated with interaction tests.

### Statistical analysis

Continuous variables are summarized as medians with interquartile ranges (IQRs), and categorical variables as counts and percentages. P/MII groups were compared using the Kruskal–Wallis test for continuous variables and the χ² test or Fisher’s exact test for categorical variables, as appropriate.

The association between P/MII and blastocysts per recorded 2PN zygote was modeled using segmented (broken-stick) Poisson regression. The log-scale linear predictor allowed one slope below an unknown breakpoint (psi) and a different slope above it. The breakpoint and its 95% CI were estimated by profile likelihood by minimizing the profile negative log-likelihood over candidate values; the confidence set was bounded using a likelihood ratio threshold of 1.92. Equality of the two slopes was tested within the same model.

Associations with embryological outcomes were estimated using Poisson rate regression with a log link, the logarithm of the recorded 2PN count as an offset, and Huber–White sandwich variance. Estimates are reported as rate ratios (RRs) with 95% CIs. Embryological models were adjusted *a priori* for age, infertility duration, total gonadotropin dose, stimulation protocol, infertility diagnosis, cycle year, number of oocytes retrieved, trigger-day E2, AFC, and AMH.

Pregnancy analyses used records with at least one embryo transferred. A record-level audit found eight disagreements between this indicator and both Cancellation rate and the linked registry cancellation flag. Four cancellation-negative records had no transferred embryo; four cancellation-positive records had one or two transferred embryos. Using the direct transfer indicator retained n=4,273 but corrected membership and event totals. Transfer day in the original models was derived from embryo type and Day-3/5/6 flags; all records were classifiable. The source-date interval differed from that derived day in two records and was used in the new restriction analyses. Logistic models reported odds ratios with 95% CIs. Primary conditional models adjusted for age, AFC, trigger-day E2, total gonadotropin dose, stimulation protocol, oocytes retrieved, transfer day, embryos transferred, and good-quality embryo transfer. Reduced-adjustment models omitted the three transfer process variables. The assumed ordering was baseline attributes, stimulation and trigger-day measurements, retrieval/MII assessment, embryo development, transfer selection, transfer characteristics, and pregnancy outcomes. This ordering is shown in **Supplementary Figure S1** and **Supplementary Table S10**. Cancellation or deferral reasons were available for 3,500 of 3,505 records without transfer and were summarized descriptively. They were not entered as baseline covariates because they describe the subsequent selection process. Neither model identifies a causal total or direct effect. The pregnancy analyses describe associations within selected transfer records, not outcomes under a fresh-transfer strategy for all retrieval cycles.

Effect modification by ovarian response label was evaluated with global likelihood ratio tests. **Supplementary Table S3** reports the prespecified unadjusted categorical interaction tests together with the adjusted interaction tests from sensitivity analyses. Additional analyses modeled progesterone, MII count, and P/MII separately and jointly; stratified by major stimulation protocol and linked fertilization method; excluded records originally classified as Day-3 fresh transfers; subtracted recorded Day-3 transferred embryos from the 2PN denominator; and repeated categorical analyses across progesterone boundary scenarios. The Day-3 subtraction used the total recorded transfer count when Day-3=1 and retained only positive resulting denominators (n=7,217); 561 records were excluded, comprising 539 zero and 22 negative denominators. Fertilization origin was unavailable for the transferred embryos, so this subtraction did not reconstruct a verified culture denominator. Fertilization method interactions used robust Wald tests after excluding the single mixed-method cycle. Propensity score overlap weighting evaluated measured common support for the embryological contrast. The original cycle-split fivefold cross-validation used the same folds to compare the base model with models adding progesterone, MII, both components, P/MII, or all three. It evaluated area under the receiver operating characteristic curve(AUC), Brier score, calibration, and decision-curve net benefit. Adding P/MII to a component model was treated as adding a derived functional term, not an independent hormonal exposure. A 1,000-resample nonparametric bootstrap assessed changepoint stability. No imputation was performed; the corrected positive-transfer models included all 4,273 records. All tests were two-sided, and P<0.05 was considered statistically significant. Primary analyses were performed using R version 4.3 (R Foundation for Statistical Computing, Vienna, Austria) with the segmented, sandwich, lmtest, and survival packages. Additional analyses used Python version 3.13.7, statsmodels version 0.14.6, and scikit-learn version 1.8.0.

New *post hoc* analyses used the expanded source fields (**Supplementary Tables S14**–**S16**). Recorded culture-positive analyses retained 5,821 cycles and fitted the same adjusted Poisson models with either log(2PN) or log(recorded culture count) offsets; the culture offset analysis was also restricted to 4,960 recorded first retrievals. Logistic sensitivities examined 4,258 date-consistent transfer records without a positive thaw count or valid thaw identifier, and 3,526 additionally recorded as first retrievals. Transfer day in these models came from the source dates; analyses retaining the original derived day gave similar results. Two component models used sandwich covariance clustered on the recorded source identifier. StratifiedGroupKFold with five folds, shuffle=True, and random_state=20260815 kept each identifier in a single fold while retaining the original within-fold preprocessing and candidate feature sets. These identifier-based procedures are sensitivity analyses, not verification of person-level identity. No source counts or outcome flags were corrected, no missing values were imputed, and AUC differences were descriptive without paired uncertainty tests. OpenAI Codex (GPT-6; OpenAI; accessed September 2026) assisted with data auditing and the preparation and execution of *post hoc* analysis code. It did not adjudicate clinical events or verify patient or embryo identity.

### Ethics

The study was approved by the Ethics Committee of Liuzhou Hospital, Guangzhou Women and Children’s Medical Center (approval no. 2026-130). The committee waived the requirement for written informed consent because the study used de-identified retrospective clinical data. The study was conducted in accordance with the Declaration of Helsinki.

## Results

### Study population

The analytic cohort contained 7,778 cycles, comprising 5,408 lower- and 2,370 higher-P/MII records. The source retrieval order field classified 6,148 as first and 1,630 as later retrievals. A total of 98,558 oocytes were retrieved, including 85,956 MII oocytes, 60,309 recorded 2PN zygotes, and 33,360 blastocysts. The original positive transfer analysis contained 4,273 records (3,036 lower and 1,237 higher P/MII), with 2,415 implantation-positive, 2,367 clinical pregnancy, and 2,016 live birth flags. Miscarriage was flagged in 331 clinical pregnancies. These are existing analytic flag totals, with source adjudication limitations described above.

**Table 1** summarizes characteristics by the exploratory changepoint. Women in the higher-P/MII group were older and had lower AMH concentrations and AFC, higher basal FSH concentrations, and fewer retrieved, MII, and 2PN oocytes. Median trigger-day progesterone was 0.42 versus 0.50 ng/ml in the lower- and higher-P/MII groups (P<0.001). Median MII count was 12 versus five (P<0.001). Infertility indications and stimulation protocols also differed substantially. Among the 4,273 positive-transfer records, the lower- and higher-P/MII groups contained 3,036 and 1,237 records, respectively. Their characteristics are reported in **Supplementary Table S1**.

**Table 1:** Baseline and cycle characteristics by the exploratory P/MII changepoint.

Characteristic	P/MII ≤0.0711 (n = 5,408)	P/MII >0.0711 (n = 2,370)	P value
Age, years	32.00 (29.00–35.00)	35.00 (31.00–38.00)	<0.001
BMI, kg/m²	22.00 (20.00–24.40)	21.60 (19.80–23.80)	<0.001
AMH, ng/ml	3.36 (2.03–5.24)	1.46 (0.75–2.74)	<0.001
AFC, n	12.00 (9.00–17.00)	7.00 (4.00–10.00)	<0.001
Infertility duration, years	3.00 (2.00–6.00)	4.00 (2.00–7.00)	<0.001
Basal E2, pg/ml	32.22 (24.43–43.23)	34.80 (25.20–49.60)	<0.001
Basal FSH, IU/L	5.37 (4.56–6.36)	6.05 (4.91–7.73)	<0.001
Basal LH, IU/L	3.29 (2.40–4.54)	2.97 (2.18–4.17)	<0.001
Basal P, ng/ml	0.23 (0.17–0.33)	0.25 (0.19–0.35)	<0.001
Stimulation duration, days	10.00 (9.00–12.00)	9.00 (8.00–11.00)	<0.001
Total gonadotropin dose, ampoules	30.00 (21.50–37.50)	30.00 (22.00–37.04)	0.544
Trigger-day E2, pg/ml	2206.81 (1415.82–3256.71)	1272.55 (627.21–2287.41)	<0.001
Trigger-day FSH, IU/L	12.43 (9.56–15.68)	15.73 (12.52–18.96)	<0.001
Trigger-day LH, IU/L	0.94 (0.67–1.54)	1.69 (0.93–3.19)	<0.001
Trigger-day P, ng/ml	0.42 (0.30–0.61)	0.50 (0.30–0.84)	<0.001
Trigger-day endometrial thickness, mm	11.80 (10.00–13.20)	10.00 (8.20–12.00)	<0.001
MII oocytes, n	12.00 (9.00–17.00)	5.00 (2.00–8.00)	<0.001
Oocytes retrieved, n	14.00 (10.00–19.00)	6.00 (3.00–10.00)	<0.001
2PN zygotes, n	8.00 (6.00–12.00)	3.00 (2.00–6.00)	<0.001
Blastocysts, n	5.00 (2.00–8.00)	0.00 (0.00–3.00)	<0.001
Available Day-3 embryos, n	8.00 (5.00–11.00)	3.00 (1.00–5.00)	<0.001
Good-quality Day-3 embryos, n	4.00 (2.00–7.00)	1.00 (1.00–3.00)	<0.001
***Type of infertility, n (%)***			*0.003*
Primary	2131 (39.4)	848 (35.8)	
Secondary	3277 (60.6)	1522 (64.2)	
***Infertility diagnosis, n (%)***			*<0.001*
Tubal factor	3816 (70.6)	1417 (59.8)	
Uterine factor	101 (1.9)	97 (4.1)	
Male factor	505 (9.3)	189 (8.0)	
Ovulatory factor	326 (6.0)	233 (9.8)	
Recurrent pregnancy loss	77 (1.4)	78 (3.3)	
Other	583 (10.8)	356 (15.0)	
***Stimulation protocol, n (%)***			*<0.001*
GnRH agonist	3552 (65.7)	797 (33.6)	
GnRH antagonist	1568 (29.0)	1104 (46.6)	
PPCOS (recorded code)	247 (4.6)	280 (11.8)	
Others (recorded category)	41 (0.8)	189 (8.0)	

Values are median (IQR) for continuous variables and n (%) for categorical variables. P values are from the Kruskal–Wallis test for continuous variables and the χ² test or Fisher’s exact test for categorical variables, as appropriate. AFC, antral follicle count; AMH, anti-Müllerian hormone; BMI, body mass index; E2, estradiol; FSH, follicle-stimulating hormone; GnRH, gonadotropin-releasing hormone; LH, luteinizing hormone; MII, metaphase II; P, progesterone; P/MII, progesterone-to-metaphase II oocyte ratio; 2PN, two pronuclei.

Bold values indicate category headings; they have no statistical significance.

### Crude embryological and pregnancy outcomes

In unadjusted analyses (**Table 2**), cycles with P/MII >0.0711 had a lower 2PN yield per retrieved oocyte (57.8% vs 61.9%), blastocysts per recorded 2PN zygote (39.9% vs 58.3%), good-quality Day-3 embryo rate (48.2% vs 49.6%), and good-quality blastocyst yield (15.7% vs 25.1%) than cycles with P/MII ≤0.0711. The proportion of available Day-3 embryos was similar between groups (90.1% vs 90.0%).

**Table 2:** Crude embryological and recorded pregnancy outcomes by the exploratory P/MII changepoint.

Outcome	P/MII ≤0.0711, n/N (%)	P/MII >0.0711, n/N (%)	P value
Embryological outcomes			
Recorded 2PN zygotes per retrieved oocyte	50458/81510 (61.9)	9851/17048 (57.8)	<0.001
Blastocysts per recorded 2PN zygote	29433/50458 (58.3)	3927/9851 (39.9)	<0.001
Available Day-3 embryos	45390/50458 (90.0)	8880/9851 (90.1)	0.584
Good-quality Day-3 embryos	25050/50458 (49.6)	4744/9851 (48.2)	0.007
Good-quality blastocysts	12648/50458 (25.1)	1545/9851 (15.7)	<0.001
Recorded pregnancy outcomes			
Implantation-positive cycle	1866/3036 (61.5)	549/1237 (44.4)	<0.001
Clinical pregnancy	1834/3036 (60.4)	533/1237 (43.1)	<0.001
Live birth	1563/3036 (51.5)	453/1237 (36.6)	<0.001
Miscarriage (per clinical pregnancy)	256/1834 (14.0)	75/533 (14.1)	0.995

Values are events/number at risk (%). The denominator is retrieved oocytes for the recorded 2PN-zygote rate and recorded 2PN zygotes for the other embryological outcomes. Recorded pregnancy outcomes use the corrected cohort defined by at least one recorded embryo transferred; miscarriage is calculated per clinical pregnancy. Pregnancy outcomes denote the existing binary record flags whose clinical verification limits are described in Outcomes.

Among positive-transfer records, P/MII >0.0711 was associated with fewer implantation-positive cycles (44.4% vs 61.5%), clinical pregnancies (43.1% vs 60.4%), and live births (36.6% vs 51.5%). The proportion of clinical pregnancies ending in miscarriage was similar in the two groups (14.1% vs 14.0%).

### Adjusted associations

After multivariable adjustment, P/MII >0.0711 remained associated with fewer blastocysts per recorded 2PN zygote (aRR, 0.796; 95% CI, 0.762–0.831; P<0.001) and a modestly lower good-quality Day-3 embryo rate (aRR, 0.959; 95% CI, 0.924–0.995; P = 0.03) (**Table 3**). When P/MII was modeled continuously, each 0.01-unit increase was associated with fewer blastocysts per recorded 2PN zygote (aRR, 0.962; 95% CI, 0.957–0.967) and a lower good-quality Day-3 embryo rate (aRR, 0.992; 95% CI, 0.988–0.996).

**Table 3:** Associations between calculated P/MII and recorded embryological outcomes.

Outcome/exposure	Crude RR (95% CI)	P	Adjusted RR (95% CI)	P
Blastocysts per recorded 2PN zygote				
Per 0.01-unit increase	0.945 (0.941–0.950)	<0.001	0.962 (0.957–0.967)	<0.001
P/MII ≤0.0711	Reference	—	Reference	—
P/MII >0.0711	0.675 (0.646–0.706)	<0.001	0.796 (0.762–0.831)	<0.001
Good-quality Day-3 embryos				
Per 0.01-unit increase	0.995 (0.992–0.999)	0.006	0.992 (0.988–0.996)	<0.001
P/MII ≤0.0711	Reference	—	Reference	—
P/MII >0.0711	0.970 (0.938–1.003)	0.07	0.959 (0.924–0.995)	0.03

Data are rate ratios (95% confidence intervals) from Poisson rate regression with a log link, the logarithm of recorded 2PN count as an offset, and Huber–White sandwich variance. Adjusted models included age, infertility duration, total gonadotropin dose, stimulation protocol, infertility diagnosis, cycle year, number of oocytes retrieved, trigger-day E2, AFC and AMH. AFC, antral follicle count; AMH, anti-Müllerian hormone; CI, confidence interval; E2, estradiol; P/MII, progesterone-to-metaphase II oocyte ratio; RR, rate ratio.

In positive-transfer records, P/MII >0.0711 was associated with lower odds of an implantation-positive cycle (aOR, 0.693; 95% CI, 0.591–0.814; P<0.001), a clinical pregnancy flag (aOR, 0.694; 95% CI, 0.591–0.815; P<0.001) and a live birth flag (aOR, 0.796; 95% CI, 0.676–0.936; P = 0.006) (**Table 4**). No clear association was observed with a miscarriage flag among records flagged as clinical pregnancies (aOR, 0.758; 95% CI, 0.547–1.050; P = 0.096). Continuous P/MII analyses showed the same direction of association for implantation-positive cycles, clinical pregnancy, and live birth.

**Table 4:** Associations between calculated P/MII and recorded pregnancy outcomes among selected transfer records.

Outcome/exposure	Crude OR (95% CI)	P	Adjusted OR (95% CI)	P
Implantation-positive cycle				
Per 0.01-unit increase	0.928 (0.913–0.942)	<0.001	0.963 (0.945–0.981)	<0.001
P/MII ≤0.0711	Reference	—	Reference	—
P/MII >0.0711	0.500 (0.438–0.572)	<0.001	0.693 (0.591–0.814)	<0.001
Clinical pregnancy				
Per 0.01-unit increase	0.926 (0.912–0.941)	<0.001	0.963 (0.945–0.981)	<0.001
P/MII ≤0.0711	Reference	—	Reference	—
P/MII >0.0711	0.496 (0.434–0.567)	<0.001	0.694 (0.591–0.815)	<0.001
Live birth				
Per 0.01-unit increase	0.934 (0.919–0.949)	<0.001	0.977 (0.958–0.995)	0.015
P/MII ≤0.0711	Reference	—	Reference	—
P/MII >0.0711	0.545 (0.475–0.624)	<0.001	0.796 (0.676–0.936)	0.006
Miscarriage				
Per 0.01-unit increase	1.006 (0.981–1.032)	0.637	0.968 (0.936–1.001)	0.060
P/MII ≤0.0711	Reference	—	Reference	—
P/MII >0.0711	1.009 (0.765–1.332)	0.947	0.758 (0.547–1.050)	0.096

Data are odds ratios (95% confidence intervals) from logistic regression in the corrected positive transfer cohort (n=4,273). Adjusted models included age, AFC, trigger-day E2, total gonadotropin dose, stimulation protocol, oocytes retrieved, transfer day, embryos transferred, and good-quality embryo transfer. Miscarriage was modeled among 2,367 clinical pregnancies. Pregnancy outcomes denote the existing binary record flags, whose clinical-verification limits are described in Outcomes.

### Component analyses and predictive performance

Component comparisons evaluated the ratio alongside its two inputs (**Supplementary Table S4**). For recorded blastocyst yield per recorded 2PN zygote, progesterone alone had an aRR of 0.997 per 0.1 ng/ml (95% CI 0.993–1.000; P = 0.074). MII count had an aRR of 1.026 per oocyte (95% CI 1.020–1.033; P<0.001). The ratio remained inversely associated when both components were included (aRR 0.920 per 0.01, 95% CI 0.910–0.931; P<0.001). For live birth, P/MII alone had an aOR of 0.977 per 0.01 (95% CI 0.958–0.995; P = 0.015). With progesterone and MII included, the ratio estimate was 0.988 (95% CI 0.961–1.015; P = 0.368). Progesterone alone had an aOR of 0.970 per 0.1 ng/ml (95% CI 0.937–1.004; P = 0.079), and MII had an aOR of 1.046 per oocyte (95% CI 1.003–1.091; P = 0.034).

Reduced-adjustment models produced stronger categorical associations than the primary conditional models: ORs were 0.641 versus 0.694 for clinical pregnancy and 0.735 versus 0.796 for live birth (**Supplementary Table S5**). In fivefold out-of-fold comparisons, the clinical pregnancy AUC was 0.602 for the base model, 0.615 after adding progesterone and MII, 0.615 after adding P/MII, and 0.616 after adding all three. The corresponding live birth AUCs were 0.618, 0.624, 0.624, and 0.625. Brier score, calibration, and decision curve differences were small (**Supplementary Table S6**). These comparisons did not establish incremental prognostic value of the ratio beyond its components.

### Associations by ovarian response phenotype

The inverse association between P/MII >0.0711 and blastocysts per recorded 2PN zygote was present across database-recorded ovarian response labels but differed in magnitude (P for interaction <0.001; **Supplementary Table S2**). The association was strongest among poor responders (aRR, 0.667; 95% CI, 0.571–0.778), followed by normal responders (aRR, 0.847; 95% CI, 0.810–0.886) and women with PCOS (aRR, 0.877; 95% CI, 0.774–0.994). There was no evidence of effect modification for the good-quality Day-3 embryo rate (P for interaction=0.43).

Phenotype-specific all-cycle denominators were unchanged. In the corrected positive-transfer cohort, lower/higher group sizes were 2,446/735 for NOR, 204/32 for PCOS, and 386/470 for POR. Clinical pregnancy events were 1,495/355, 118/13, and 221/165, respectively; live birth events were 1,290/311, 96/12, and 177/130. The corrected unadjusted interaction P values were 0.062 for clinical pregnancy and 0.078 for live birth; adjusted interaction sensitivity P values were 0.263 and 0.310. These results do not support phenotype-specific claims for recorded pregnancy outcomes.

For recorded pregnancy outcomes, the corrected unadjusted global interaction P value was 0.026 for implantation-positive cycle (**Supplementary Table S3**). The corresponding unadjusted interaction P values were 0.062 for clinical pregnancy and 0.078 for live birth. Adjusted sensitivity interaction P values were 0.149, 0.263, and 0.310, respectively. These tests did not support robust phenotype-specific conclusions for clinical outcomes. Within-phenotype estimates are reported in **Supplementary Table S3**.

### Sensitivity analyses and transfer selection

Existing protocol-stratified sensitivities are reported in **Supplementary Table S7**. For recorded blastocyst yield, aRRs were 0.831 (95% CI 0.787–0.877) in agonist cycles and 0.749 (0.691–0.812) in antagonist cycles. Reduced-adjustment live birth aORs were 0.702 (0.573–0.861) and 0.815 (0.629–1.056), respectively. Measured exposure group overlap was broad in the embryological cohort: 7,754 of 7,775 complete cases were within empirical common support. The overlap-weighted recorded blastocyst RR was 0.836 (95% CI 0.788–0.887). Positive-transfer proportions were 56.1% in lower- and 52.2% in higher-P/MII records. They varied by protocol: 2,821/4,349 agonist, 1,400/2,672 antagonist, 52/230 other, and 0/527 PPCOS-coded cycles had a positive recorded transfer count. This category contributed no records to the pregnancy models. The absence of observed transfers limits empirical support for transport to that category; the available fields do not establish either a PPOS protocol mapping or a protocol-defined structural zero. Inverse-probability-of-selection weighting was not used to claim such transport. The recorded blastocyst association persisted after excluding Day-3 transfer cycles (aRR 0.861, 95% CI 0.824–0.900) and subtracting recorded Day-3 transferred embryos (aRR 0.858, 0.825–0.892). These checks did not account fully for cleavage-stage cryopreservation or recover a verified extended culture denominator. Progesterone boundary sensitivities were materially unchanged (**Supplementary Tables S7**, **S8**, **S11**).

Registry linkage was complete (7,778/7,778). Additional adjustment for fertilization method left the main associations essentially unchanged (**Supplementary Table S13**). The inverse recorded blastocyst association was observed in conventional IVF (aRR 0.779, 95% CI 0.738–0.821) and ICSI-based cycles (aRR 0.845, 0.786–0.908). Its magnitude differed by method (P for interaction=0.003). Clinical pregnancy and live birth interactions were not detected (P = 0.972 and P = 0.935). Among 3,505 records without transfer, 3,500 had a linked reason. Common reasons were high OHSS risk (n=1,409), PGT testing (n=602), illness or personal reasons (n=398), hydrosalpinx (n=318), and PGT pending (n=207). Thirty-seven records had no transferable embryo. These categories described differing clinical and management pathways, rather than a single elective freeze-all population.

### Source-linked *post hoc* sensitivity analyses

Among 5,821 records with positive culture counts, recorded blastocyst-to-culture totals were 29,433/46,428 (63.4%) in the lower group and 3,927/6,529 (60.1%) in the higher group. On this identical population, high-versus-low adjusted rate ratios were 0.934 (95% CI 0.902–0.968; P<0.001) with the 2PN offset and 0.974 (0.945–1.005; P = 0.101) with the culture-count offset. The continuous culture-count rate ratio was 0.993 per 0.01 increase (0.989–0.997; P<0.001). The 4,960 first-retrieval culture records had no thawing evidence; categorical and continuous estimates were 0.973 (0.940–1.006; P = 0.108) and 0.993 (0.989–0.997; P<0.001), respectively (**Supplementary Table S14**). Thus, the categorical comparison became uncertain, while the continuous association remained small.

Source-restricted transfer analyses included 4,258 records without thawing evidence, of which 3,526 were recorded first retrievals. After simultaneous inclusion of P and MII, the ratio term in the first-retrieval subset was associated with clinical pregnancy flags (aOR 0.952 per 0.01, 95% CI 0.919–0.986; P = 0.006), but it was uncertain for live birth flags (0.968, 0.935–1.002; P = 0.067). In the full no-thaw subset, corresponding estimates were 0.973 (0.946–1.001; P = 0.059) and 0.990 (0.963–1.018; P = 0.494). Results using the original transfer-day derivation were similar (**Supplementary Table S15**).

Grouping records on the 3,912 distinct source identifiers in the original 4,273-record transfer cohort gave component-adjusted ratio estimates of 0.971 (95% CI 0.945–0.997; P = 0.030) for clinical pregnancy and 0.988 (0.961–1.015; P = 0.367) for live birth. Grouped-fold live birth AUCs were 0.6232 for Base+P+MII, 0.6228 for Base+P/MII, and 0.6243 for the combined model. No source identifier appeared in both training and test sets within a fold (**Supplementary Table S16**). These small descriptive performance differences do not establish clinical benefit.

### Exploratory P/MII changepoint

Segmented Poisson regression identified an exploratory changepoint of 0.0711 ng/ml per MII oocyte (95% profile likelihood CI 0.0541–0.0861; **Figure 1**). The exact value is retained to reproduce the reported categorical analyses; it does not define a clinical threshold. In 1,000 bootstrap resamples of a reconstructed adjusted segmented model, the median was 0.071 and the 2.5th–97.5th percentiles were 0.055–0.086. The reconstructed full-sample estimate was 0.0687, rather than exactly reproducing the original R estimate. These results describe internal stability around 0.07, not external validity or four-decimal clinical precision (**Supplementary Table S9**).

**Figure 1:**
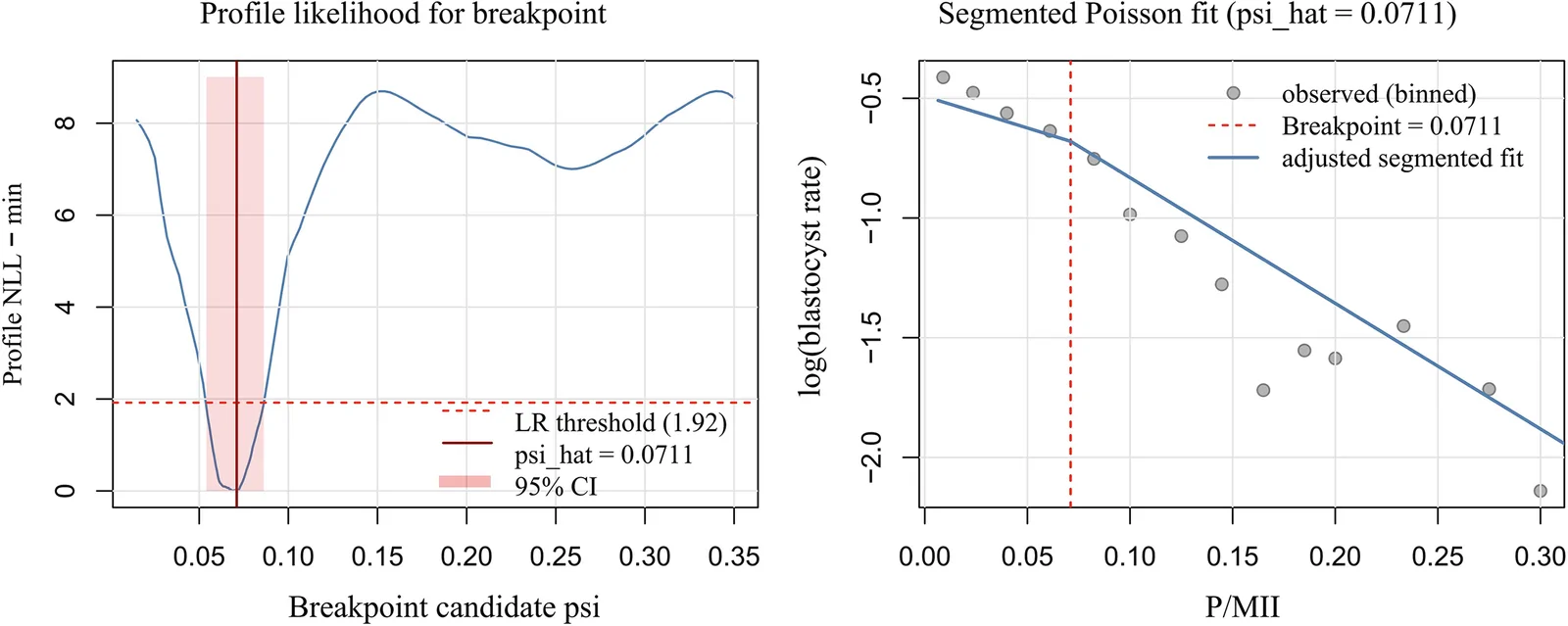
Exploratory segmented Poisson analysis of calculated P/MII and cycle-level recorded blastocyst yield per recorded 2PN zygote. Left: Profile likelihood across candidate changepoints; the estimate was 0.0711 (95% profile likelihood CI 0.0541–0.0861). The dashed line marks the likelihood ratio threshold of 1.92. Right: Observed log-yield, binned by P/MII decile, and the adjusted segmented fit. The fitted slopes differed (P<0.001). The plotted “blastocyst rate” denotes the recorded cycle-level yield, whose denominator includes Day-3 transferred and cleavage-stage cryopreserved embryos. The changepoint is an exploratory estimate and should not be used as a clinical cutoff. P/MII uses trigger-day progesterone concentration divided by the subsequently recorded MII count. CI, confidence interval; LR, likelihood ratio; NLL, negative log-likelihood.

## Discussion

In this single-center cohort of 7,778 IVF/ICSI cycle records, higher P/MII was associated with lower recorded cycle-level blastocyst yield per recorded 2PN zygote and a small reduction in good-quality Day-3 embryo yield. Among positive transfer records, higher P/MII was associated with lower odds of an implantation-positive cycle, clinical pregnancy and live birth flags, with no clear association with a miscarriage flag among records flagged as clinical pregnancies. Associations were also observed in continuous models. These findings require qualification: the live birth association was not evident when progesterone and MII count were included with the ratio, and the prediction comparisons did not establish incremental value beyond those components. Embryological associations varied across database-recorded ovarian response labels, whereas corrected interaction tests did not support phenotype-specific conclusions for clinical pregnancy or live birth. The internally derived value of 0.0711 is retained for analytic reproducibility and is not a validated clinical cutoff. The newly available culture-count and source-restricted results further qualify the magnitude and interpretation of these original associations.

The ratio’s dependence on its denominator is central to interpretation. In **Table 1**, median trigger-day progesterone was 0.42 versus 0.50 ng/ml in lower- versus higher-P/MII groups (P<0.001), whereas median MII count was 12 versus 5 (P<0.001). Thus, the groups differed in progesterone concentration as well as markedly in ovarian response; these comparisons do not quantify the relative contribution of each component to the outcome association. In the component models, the ratio remained associated with the recorded blastocyst endpoint but not with live birth after simultaneous inclusion of progesterone and MII count (aOR 0.988 per 0.01, 95% CI 0.961–1.015; P = 0.368). Because P/MII is a deterministic function of its components, adding it to a component model introduces a particular nonlinear function, not an independently manipulable hormonal exposure. These results do not establish an independent hormonal effect or incremental prognostic value. MII ascertainment also differed by fertilization method. Associations in both conventional IVF and ICSI-based strata do not remove residual differences in maturity assessment, sperm factors, laboratory practice, or treatment selection. Component-adjusted clinical pregnancy associations remained in the identifier-clustered and first-retrieval sensitivities; absence of a live birth signal must not be generalized to every outcome.

The prediction comparisons reinforce a restrained clinical interpretation. The original cycle-split cross-validation gave modest discrimination but did not keep shared source identifiers in the same fold. The new identifier-grouped analyses used five folds without any identifier overlap between training and test sets. Clinical pregnancy AUCs ranged from 0.604 to 0.619 and live birth AUCs from 0.617 to 0.624. For live birth, adding both components, P/MII, or all three to the base model gave 0.623, 0.623, and 0.624, respectively. These small descriptive differences lack paired uncertainty estimates and do not establish superiority or clinical usefulness. Grouping the recorded identifiers does not itself validate their patient-level meaning or provide external validation. No PPCOS-coded record had a positive transfer count, so pregnancy models cannot be extrapolated to that category or to a strategy of fresh transfer for all retrievals. The findings do not support using P/MII for trigger-day decisions, a freeze-all rule, or individual clinical counseling.

P/MII is a derived statistical measure rather than a measured hormone exposure. Progesterone was measured on the trigger day, whereas the mature oocyte count and the ratio became available after retrieval. Dividing progesterone concentration by MII count does not establish a physiological measure of endometrial exposure or receptivity. Evidence concerning absolute progesterone and endometrial changes (13, 14), circulating progesterone and pregnancy outcomes (24), or follicular-fluid steroid concentrations and fertilization (25) concerns exposures distinct from this ratio and cannot validate a ratio-specific mechanism. We did not measure endometrial maturation, follicular-fluid hormones, or relevant molecular pathways. The present data consequently do not distinguish hormonal biology from ovarian response, treatment selection, or culture and transfer practices.

The exploratory changepoint should be considered alongside the heterogeneous ratio thresholds reported previously. Singh et al. reported a progesterone/oocyte threshold of 0.15 using a receiver-operating-characteristic approach for pregnancy in 687 fresh long-agonist IVF/ICSI cycles (18). Simon et al. observed lower implantation and live birth rates in the highest PMOI category, at >=0.167 ng/ml per mature oocyte, among 960 first fresh ICSI transfers after recombinant FSH/GnRH-antagonist stimulation (20). Sun et al. reported a PMOI value of 0.063 using ROC/Youden analysis for clinical pregnancy in 1,239 fresh IVF/ICSI cycles treated with a fixed GnRH-antagonist regimen (19). Huang et al. reported an inflection point of 0.367 using smooth-curve/two-piecewise modeling for clinical pregnancy in 3,566 fresh ICSI cycles with normal ovarian response (17). Differences in populations, stimulation regimens, assays, denominator ascertainment, endpoints, and threshold-selection methods limit direct comparison. Our segmentation endpoint was recorded blastocyst yield per recorded 2PN zygote, rather than pregnancy. Numerical proximity to a previous threshold therefore does not constitute validation. The wide between-study range, the present profile likelihood interval, and previous work on denominator dependence (26) argue against assigning a universal biological or clinical meaning to the ratio threshold.

The blastocyst endpoint depends strongly on the observed culture pathway. The original 2PN denominator included cleavage-stage transfer and cryopreservation, so it combined embryo development with embryo allocation. In the expanded source, positive culture counts were recorded in 4,734 of 5,408 lower-P/MII cycles and 1,087 of 2,370 higher-P/MII cycles. Their recorded blastocyst-to-culture totals were 63.4% and 60.1%. Within the same culture-positive population, the categorical adjusted association attenuated when the denominator changed from recorded 2PN to recorded culture count, although a small continuous association remained. The first-retrieval culture analysis, with no recorded thawing evidence, showed a similar pattern. These findings support an important role for culture population and denominator choice; they do not establish that all association is mathematical or that embryo developmental potential is unaffected. Culture selection, heterogeneous laboratory pathways, and lack of embryo-level fertilization provenance remain relevant. Previous reports of variation by the developmental stage of transferred embryos also underscore the importance of the transfer context (27, 28).

### Strengths and limitations

The study has several strengths: a large cycle-level cohort, complete linkage to expanded source fields, embryological and clinical outcomes in the same population, a single progesterone assay, continuous and categorical modeling of P/MII, profile likelihood estimation of the exploratory changepoint, and formal interaction testing across ovarian response labels. Poisson rate regression with a recorded 2PN offset and robust variance was used to estimate embryological rate ratios rather than odds ratios. These features support within-cohort estimation, but they do not establish causal, predictive, or threshold validity.

The retrospective single-center design and heterogeneous stimulation protocols, infertility indications, and ovarian response profiles leave residual and unmeasured confounding. Adjustment, stratification, and source-based restrictions do not make these treatment pathways comparable. Medication-level details were unavailable for classifying the high-progestogen source category as a standardized PPOS regimen. Because transfer selection and transfer characteristics occur after retrieval, the pregnancy models estimate associations conditional on selected treatment records rather than causal effects of progesterone, MII yield, or their ratio. Cancellation or deferral reasons are part of this selection process rather than interchangeable baseline confounders. The changepoint was derived in the same cohort and is not a validated clinical threshold. Flexible nonlinear component comparisons, paired uncertainty estimates for performance differences, and external validation were not performed.

The blastocyst endpoint has a further limitation arising from embryo provenance. The recorded culture denominator improves on the original 2PN denominator but does not establish a 2PN-specific biological blastulation rate. Individual cultured embryos and blastocysts were not linked to pronuclear or retrieval origins. All 173 records with blastocyst counts exceeding 2PN counts were compatible with their recorded culture counts. All 22 negative Day-3 subtraction records had zero culture counts, and 23 zero-subtraction records with blastocysts had positive culture counts, showing why subtraction cannot reliably reconstruct culture allocation. The full culture-positive population included 132 thaw-positive records, whereas the 4,960 recorded first-retrieval culture records had no thawing evidence. These findings support recorded culture counts as a sensitivity denominator without verifying individual embryo origins. Differences in MII ascertainment between fertilization methods, unmeasured laboratory or sperm factors, and boundary-value progesterone substitutions also remain relevant.

Pregnancy analyses were restricted to 4,273 positive transfer records and do not represent a fresh-transfer strategy for all 7,778 retrievals. The 3,505 records without transfer followed heterogeneous clinical pathways; none of the 527 records labelled stimulation under high progestogen levels contributed a positive transfer count. Although all positive transfer intervals were 3–6 days, 15 records also contained thawing evidence. Eight cancellation flags conflicted with transfer counts. The original cohort included later retrievals and repeated source identifiers; identifier-based clustering and grouped validation address observed grouping without independently establishing patient identity. Existing outcome flags were preserved rather than re-adjudicated, and incomplete ultrasound or gestational-age information limits verification of the clinical criteria described in Methods. Missing loss weeks in 149 miscarriage records, two recorded losses at 24 weeks or later, and missing delivery gestational age in 749 live birth records mean that the intended clinical definitions were not verified for all flags. Outcome misclassification may therefore affect the estimates. Sequential eligibility and uterine/cavity exclusions were not reconstructable, and complete phenotype ascertainment was unavailable. Cumulative live birth, subsequent frozen-transfer outcomes, neonatal outcomes, and mechanistic biomarkers were unavailable.

## Conclusions

In this retrospective cohort, P/MII was associated with recorded embryo output and selected pregnancy outcome flags, but the magnitude and evidence differed with the culture population, denominator, and outcome. The categorical recorded culture association was uncertain, while a small continuous association remained. Clinical pregnancy associations persisted in some component-adjusted sensitivities; incremental clinical usefulness and an independent physiological ratio effect were not established. P/MII is calculated after retrieval and is not a direct measure of endometrial progesterone exposure. The findings do not support a clinical cutoff, trigger-day decision marker, or freeze-all rule. Future studies should evaluate comparable treatment populations using embryo-level provenance, clinically adjudicated endpoints, flexible component modeling, and independent validation.

## Data Availability

The original contributions presented in the study are included in the article/**Supplementary Material**. Additional data are available from the corresponding authors upon reasonable request, subject to approval by the institutional ethics committee and applicable data-protection requirements.
