# Supplementary material for: Trigger-day progesterone-to-mature-oocyte ratio, cycle-level blastocyst yield, and recorded embryo-transfer outcomes: a retrospective cohort study
Source: Front Endocrinol (Lausanne). 2026 Oct 28;17:1938120. doi: 10.3389/fendo.2026.1938120 (PMC13752009; doi:10.3389/fendo.2026.1938120)
Supplement: Supplementary file 1 [file SupplementaryFile1.docx]

**Supplementary material**

P/MII denotes trigger-day serum progesterone divided by the MII-oocyte count recorded after retrieval; the ratio becomes available after retrieval and maturity assessment. The original recorded blastocyst yield denotes cycle-level blastocyst count per recorded 2PN zygote. Table S14 separately evaluates recorded blastocyst yield per recorded cultured embryo. Neither is a verified 2PN-specific biological blastulation rate. The original pregnancy analyses use records with a positive embryo-transfer count; new date, thawing and retrieval-order restrictions are described in Table S15. Pregnancy outcomes throughout the tables denote existing binary record flags, with source-verification limits described in the manuscript Outcomes and Strengths and limitations.

**Table S1. Baseline and cycle characteristics of selected positive-transfer records by calculated P/MII group (n = 4,273).**

| **Characteristic** | **P/MII ≤0.0711 (n = 3,036)** | **P/MII >0.0711 (n = 1,237)** | ***P* value** |
| --- | --- | --- | --- |
| Age, years | 32.50 (29.00-36.00) | 34.00 (31.00-37.00) | <0.001 |
| BMI, kg/m² | 22.00 (20.00-24.50) | 21.60 (19.80-23.80) | <0.001 |
| AMH, ng/mL | 2.92 (1.87-4.38) | 1.62 (0.90-2.85) | <0.001 |
| AFC, n | 11.00 (8.00-15.00) | 8.00 (5.00-11.00) | <0.001 |
| Infertility duration, years | 3.00 (2.00-6.00) | 4.00 (2.00-7.00) | <0.001 |
| Basal E2, pg/mL | 31.76 (24.29-43.20) | 34.16 (25.01-47.65) | <0.001 |
| Basal FSH, IU/L | 5.53 (4.69-6.51) | 5.94 (4.88-7.37) | <0.001 |
| Basal LH, IU/L | 3.17 (2.36-4.30) | 2.90 (2.15-3.99) | <0.001 |
| Basal P, ng/mL | 0.23 (0.17-0.32) | 0.25 (0.19-0.35) | <0.001 |
| Trigger-day E2, pg/mL | 1825.67 (1241.63-2558.21) | 1300.61 (706.89-2077.29) | <0.001 |
| Trigger-day FSH, IU/L | 13.14 (10.28-16.11) | 15.95 (12.91-18.94) | <0.001 |
| Trigger-day LH, IU/L | 0.89 (0.65-1.31) | 1.35 (0.84-2.58) | <0.001 |
| Trigger-day P, ng/mL | 0.40 (0.27-0.53) | 0.53 (0.34-0.80) | <0.001 |
| Trigger-day endometrial thickness, mm | 11.80 (10.00-13.30) | 10.80 (9.20-12.40) | <0.001 |
| ***Infertility diagnosis, n (%)*** |  |  | <0.001 |
| Tubal factor | 2309 (76.1) | 853 (69.0) |  |
| Uterine factor | 52 (1.7) | 39 (3.2) |  |
| Male factor | 342 (11.3) | 139 (11.2) |  |
| Ovulatory factor | 168 (5.5) | 97 (7.8) |  |
| Recurrent pregnancy loss | 1 (0.0) | 0 (0.0) |  |
| Other | 164 (5.4) | 109 (8.8) |  |
| ***Type of infertility, n (%)*** |  |  | 0.358 |
| Primary | 1174 (38.7) | 459 (37.1) |  |
| Secondary | 1862 (61.3) | 778 (62.9) |  |
| ***Stimulation protocol, n (%)*** |  |  | <0.001 |
| GnRH agonist | 2245 (73.9) | 576 (46.6) |  |
| GnRH antagonist | 785 (25.9) | 615 (49.7) |  |
| Other protocols | 6 (0.2) | 46 (3.7) |  |
| ***Transferred good-quality embryos, n (%)*** |  |  | <0.001 |
| No | 480 (15.8) | 324 (26.2) |  |
| Yes | 2556 (84.2) | 913 (73.8) |  |
| ***Day of embryo transfer, n (%)*** |  |  | <0.001 |
| Day 3 | 1763 (58.1) | 1066 (86.2) |  |
| Day 5 | 1235 (40.7) | 161 (13.0) |  |
| Day 6 | 38 (1.3) | 10 (0.8) |  |

Values are median (IQR) for continuous variables and n (%) for categorical variables. P values are from the Kruskal–Wallis test for continuous variables and from the χ² test or Fisher’s exact test for categorical variables, as appropriate. AFC, antral follicle count; AMH, anti-Müllerian hormone; BMI, body mass index; E2, estradiol; FSH, follicle-stimulating hormone; GnRH, gonadotropin-releasing hormone; LH, luteinizing hormone; P, progesterone; P/MII, progesterone-to-metaphase II oocyte ratio; In the full 7,778-record cohort, all 527 PPCOS-coded records map to the source description stimulation under high progestogen levels; specific drugs and a standardized PPOS regimen remain unverified. Percentages may not sum to 100% because of rounding. In that full cohort, the pooled Other protocols category comprises mild stimulation (183), luteal-phase stimulation (44), and natural cycles (three). No PPCOS-coded record had a positive transfer count (0/527; Table S11).

**Table S2. Embryological subgroup analyses by ovarian-response phenotype (Poisson rate regression with robust variance).**

| **Subgroup** | **P/MII group** | **Crude RR (95% CI)** | ***P*** | **Adjusted RR (95% CI)** | ***P*** | ***P* interaction** |
| --- | --- | --- | --- | --- | --- | --- |
| **Recorded blastocyst yield** |  |  |  |  |  | <0.001 |
| NOR | P/MII ≤0.0711 | Reference | — | Reference | — |  |
| NOR | P/MII >0.0711 | 0.778 (0.744–0.813) | <0.001 | 0.847 (0.810–0.886) | <0.001 |  |
| PCOS | P/MII ≤0.0711 | Reference | — | Reference | — |  |
| PCOS | P/MII >0.0711 | 0.863 (0.761–0.980) | 0.02 | 0.877 (0.774–0.994) | 0.04 |  |
| POR | P/MII ≤0.0711 | Reference | — | Reference | — |  |
| POR | P/MII >0.0711 | 0.471 (0.409–0.543) | <0.001 | 0.667 (0.571–0.778) | <0.001 |  |
| ***Good-quality Day-3 embryo yield*** |  |  |  |  |  | 0.43 |
| NOR | P/MII ≤0.0711 | Reference | — | Reference | — |  |
| NOR | P/MII >0.0711 | 0.967 (0.929–1.007) | 0.10 | 0.954 (0.914–0.997) | 0.03 |  |
| PCOS | P/MII ≤0.0711 | Reference | — | Reference | — |  |
| PCOS | P/MII >0.0711 | 0.946 (0.800–1.118) | 0.51 | 0.945 (0.799–1.118) | 0.51 |  |
| POR | P/MII ≤0.0711 | Reference | — | Reference | — |  |
| POR | P/MII >0.0711 | 1.017 (0.946–1.092) | 0.65 | 0.955 (0.880–1.037) | 0.28 |  |

Data are rate ratios (95% confidence intervals) from Poisson rate regression with a log link, the logarithm of recorded 2PN count as an offset, and robust sandwich variance. Adjusted models included age, infertility duration, total gonadotropin dose, ovarian stimulation protocol, infertility diagnosis, cycle year, number of oocytes retrieved, trigger-day E2, AFC and AMH; the stratifying variable (ovarian-response phenotype) was excluded from each within-stratum model. P interaction is from a likelihood-ratio test of the P/MII × phenotype interaction term. CI, confidence interval; NOR, normal ovarian responder; PCOS, polycystic ovary syndrome; POR, poor ovarian responder; RR, rate ratio.

**Table S3. Pregnancy-outcome subgroup analyses among selected positive-transfer records by ovarian-response phenotype (logistic regression).**

| **Subgroup** | **P/MII group** | **Crude OR (95% CI)** | ***P*** | **Adjusted OR (95% CI)** | ***P*** | ***P* interaction** |
| --- | --- | --- | --- | --- | --- | --- |
| ***Implantation-positive cycle*** |  |  |  |  |  | 0.026 |
| NOR | P/MII ≤0.0711 | Reference | — | Reference | — |  |
| NOR | P/MII >0.0711 | 0.610 (0.516-0.720) | <0.001 | 0.744 (0.615-0.900) | 0.002 |  |
| PCOS | P/MII ≤0.0711 | Reference | — | Reference | — |  |
| PCOS | P/MII >0.0711 | 0.469 (0.220-1.002) | 0.051 | 0.436 (0.185-1.028) | 0.058 |  |
| POR | P/MII ≤0.0711 | Reference | — | Reference | — |  |
| POR | P/MII >0.0711 | 0.393 (0.298-0.519) | <0.001 | 0.666 (0.477-0.928) | 0.016 |  |
| ***Clinical pregnancy*** |  |  |  |  |  | 0.062 |
| NOR | P/MII ≤0.0711 | Reference | — | Reference | — |  |
| NOR | P/MII >0.0711 | 0.594 (0.503-0.702) | <0.001 | 0.735 (0.608-0.889) | 0.001 |  |
| PCOS | P/MII ≤0.0711 | Reference | — | Reference | — |  |
| PCOS | P/MII >0.0711 | 0.499 (0.234-1.064) | 0.072 | 0.456 (0.194-1.076) | 0.073 |  |
| POR | P/MII ≤0.0711 | Reference | — | Reference | — |  |
| POR | P/MII >0.0711 | 0.404 (0.306-0.533) | <0.001 | 0.686 (0.492-0.956) | 0.026 |  |
| ***Live birth*** |  |  |  |  |  | 0.078 |
| NOR | P/MII ≤0.0711 | Reference | — | Reference | — |  |
| NOR | P/MII >0.0711 | 0.657 (0.556-0.776) | <0.001 | 0.844 (0.697-1.021) | 0.080 |  |
| PCOS | P/MII ≤0.0711 | Reference | — | Reference | — |  |
| PCOS | P/MII >0.0711 | 0.675 (0.314-1.453) | 0.315 | 0.656 (0.269-1.601) | 0.355 |  |
| POR | P/MII ≤0.0711 | Reference | — | Reference | — |  |
| POR | P/MII >0.0711 | 0.451 (0.340-0.600) | <0.001 | 0.727 (0.514-1.030) | 0.073 |  |

Data are crude and adjusted odds ratios (95% confidence intervals) from logistic regression among positive-transfer records. Adjusted models included age, AFC, trigger-day E2, total gonadotropin dose, ovarian stimulation protocol, number of oocytes retrieved, day of embryo transfer, number of embryos transferred and transfer of a good-quality embryo; the stratifying variable (ovarian-response phenotype) was excluded from each within-stratum model. The displayed global P interaction values are from the prespecified unadjusted categorical likelihood-ratio tests; adjusted sensitivity interaction P values were 0.149 for implantation-positive cycle, 0.263 for clinical pregnancy, and 0.310 for live birth. CI, confidence interval; NOR, normal ovarian responder; OR, odds ratio; PCOS, polycystic ovary syndrome; POR, poor ovarian responder.

**Supplementary statistical methods**

Additional analyses in Tables S4-S16 and Supplementary Figure S1 evaluated robustness, model specification, temporal ordering, source-registry linkage, and data integrity. Corrected positive-transfer analyses defined transfer by a positive number of embryos transferred. Primary conditional models included transfer-process variables, whereas reduced-adjustment models omitted them. Supplementary Figure S1 and Table S10 state the assumed ordering and analytic role of measured variables. Neither model is interpreted as a causal total or direct effect because P/MII combines trigger-day progesterone with a post-retrieval MII denominator and pregnancy analyses condition on transfer selection. The original embryological models used the logarithm of recorded 2PN count as an offset and cycle-level robust variance; the new culture-denominator analyses are specified in Table S14.

Fivefold stratified cross-validation used the same folds for each candidate model, with a fixed random seed of 20260815. The base model included age, AFC, trigger-day E2, total gonadotropin dose, number of oocytes retrieved, and stimulation protocol. Continuous predictors were standardized within each training fold and stimulation protocol was one-hot encoded. Performance was summarized by out-of-fold AUC, Brier score, and calibration intercept and slope. Decision-curve net benefit was evaluated from threshold probabilities 0.20 to 0.80. These original folds were split by cycle. The new source-identifier-grouped validation and clustered component-model sensitivities are reported separately in Table S16.

For overlap weighting of the all-cycle embryological analysis, the propensity score included measured baseline, stimulation, trigger-day, infertility, protocol, and calendar variables. Transfer-selection weighting to the complete retrieval cohort was not performed because no positive transfer was observed among PPCOS-coded records (0/527); this provides no empirical support for that category but does not establish protocol-defined structural non-transfer. The linked source registry provided cancellation or transfer-deferral reasons for 3,500/3,505 cycles without a recorded transfer; these post-retrieval reasons were summarized in Table S13 rather than treated as baseline confounders. Day-3 sensitivities excluded records originally classified as Day-3 fresh transfers and, separately, subtracted the recorded number of Day-3 transferred embryos from the 2PN denominator. These original proxy analyses did not reconstruct the extended-culture population. The newly linked recorded culture count is used directly in Table S14, with embryo-provenance limitations retained.

No imputation was performed. The corrected primary conditional, reduced-adjustment, and prediction analyses included all 4,273 positive-transfer records. The cohort audit excluded four records with no transferred embryo and included four records with one or two transferred embryos, leaving the total sample size unchanged at 4,273. A 1,000-resample bootstrap was performed for changepoint stability. New source-linked sensitivities preserve those existing flags and counts and use the additional restrictions described in Tables S14-S16.

**Table S4. Component and joint models addressing mathematical coupling.**

| **Outcome** | **Model term** | **Effect** | **Estimate (95% CI)** | **P** | **n** |
| --- | --- | --- | --- | --- | --- |
| Recorded blastocyst yield | P alone, per 0.1 ng/mL | aRR | 0.997 (0.993-1.000) | 0.074 | 7,778 |
| Recorded blastocyst yield | MII alone, per 1 oocyte | aRR | 1.026 (1.020-1.033) | <0.001 | 7,778 |
| Recorded blastocyst yield | P/MII alone, per 0.01 | aRR | 0.962 (0.957-0.967) | <0.001 | 7,778 |
| Recorded blastocyst yield | P/MII with P and MII, per 0.01 | aRR | 0.920 (0.910-0.931) | <0.001 | 7,778 |
| Clinical pregnancy | P alone, per 0.1 ng/mL | aOR | 0.956 (0.922-0.991) | 0.015 | 4,273 |
| Clinical pregnancy | MII alone, per 1 oocyte | aOR | 1.049 (1.006-1.093) | 0.025 | 4,273 |
| Clinical pregnancy | P/MII alone, per 0.01 | aOR | 0.963 (0.945-0.981) | <0.001 | 4,273 |
| Clinical pregnancy | P/MII with P and MII, per 0.01 | aOR | 0.971 (0.945-0.997) | 0.030 | 4,273 |
| Live birth | P alone, per 0.1 ng/mL | aOR | 0.970 (0.937-1.004) | 0.079 | 4,273 |
| Live birth | MII alone, per 1 oocyte | aOR | 1.046 (1.003-1.091) | 0.034 | 4,273 |
| Live birth | P/MII alone, per 0.01 | aOR | 0.977 (0.958-0.995) | 0.015 | 4,273 |
| Live birth | P/MII with P and MII, per 0.01 | aOR | 0.988 (0.961-1.015) | 0.368 | 4,273 |

**Table S5. Reduced-adjustment and primary conditional models among selected positive-transfer records.**

| **Outcome** | **Model** | **Adjusted OR (95% CI)** | **P** | **n** |
| --- | --- | --- | --- | --- |
| Implantation-positive cycle | Reduced-adjustment model | 0.640 (0.548-0.749) | <0.001 | 4,273 |
| Implantation-positive cycle | Primary conditional model | 0.693 (0.591-0.814) | <0.001 | 4,273 |
| Clinical pregnancy | Reduced-adjustment model | 0.641 (0.548-0.749) | <0.001 | 4,273 |
| Clinical pregnancy | Primary conditional model | 0.694 (0.591-0.815) | <0.001 | 4,273 |
| Live birth | Reduced-adjustment model | 0.735 (0.627-0.862) | <0.001 | 4,273 |
| Live birth | Primary conditional model | 0.796 (0.676-0.936) | 0.006 | 4,273 |

**Table S6. Fivefold out-of-fold predictive performance.**

| **Outcome** | **Model** | **n** | **Events** | **AUC** | **Brier** | **Cal. intercept** | **Cal. slope** |
| --- | --- | --- | --- | --- | --- | --- | --- |
| Clinical pregnancy | Base | 4,273 | 2,367 | 0.602 | 0.238 | 0.015 | 0.930 |
| Clinical pregnancy | Base + P | 4,273 | 2,367 | 0.608 | 0.237 | 0.019 | 0.912 |
| Clinical pregnancy | Base + MII | 4,273 | 2,367 | 0.609 | 0.237 | 0.015 | 0.931 |
| Clinical pregnancy | Base + P + MII | 4,273 | 2,367 | 0.615 | 0.237 | 0.018 | 0.915 |
| Clinical pregnancy | Base + P/MII | 4,273 | 2,367 | 0.615 | 0.236 | 0.016 | 0.931 |
| Clinical pregnancy | Base + P + MII + P/MII | 4,273 | 2,367 | 0.616 | 0.236 | 0.019 | 0.915 |
| Live birth | Base | 4,273 | 2,016 | 0.618 | 0.238 | -0.005 | 0.956 |
| Live birth | Base + P | 4,273 | 2,016 | 0.619 | 0.238 | -0.005 | 0.952 |
| Live birth | Base + MII | 4,273 | 2,016 | 0.623 | 0.237 | -0.005 | 0.956 |
| Live birth | Base + P + MII | 4,273 | 2,016 | 0.624 | 0.237 | -0.006 | 0.951 |
| Live birth | Base + P/MII | 4,273 | 2,016 | 0.624 | 0.237 | -0.005 | 0.952 |
| Live birth | Base + P + MII + P/MII | 4,273 | 2,016 | 0.625 | 0.237 | -0.007 | 0.940 |

Decision-curve analysis across threshold probabilities 0.20-0.80 showed only small differences among the base, component, P/MII, and combined models.

**Table S7. Protocol, Day-3, overlap-weighted, and progesterone reporting-boundary sensitivity analyses.**

| **Sensitivity analysis** | **Adjusted estimate (95% CI)** | **P** | **n** |
| --- | --- | --- | --- |
| Recorded blastocyst yield: Agonist | 0.831 (0.787-0.877) | <0.001 | 4,349 |
| Clinical pregnancy: Agonist, reduced-adjustment covariates | 0.637 (0.519-0.781) | <0.001 | 2,821 |
| Live birth: Agonist, reduced-adjustment covariates | 0.702 (0.573-0.861) | <0.001 | 2,821 |
| Recorded blastocyst yield: Antagonist | 0.749 (0.691-0.812) | <0.001 | 2,672 |
| Clinical pregnancy: Antagonist, reduced-adjustment covariates | 0.660 (0.514-0.846) | 0.001 | 1,400 |
| Live birth: Antagonist, reduced-adjustment covariates | 0.815 (0.629-1.056) | 0.121 | 1,400 |
| Recorded blastocyst yield, excluding records originally classified as Day-3 fresh transfers | 0.861 (0.824-0.900) | <0.001 | 4,949 |
| Recorded blastocyst yield after subtracting recorded Day-3 transferred embryos from the recorded 2PN denominator | 0.858 (0.825-0.892) | <0.001 | 7,217 |
| Overlap-weighted recorded blastocyst yield | 0.836 (0.788-0.887) | <0.001 | 7,775 |
| Recorded blastocyst yield: Exclude records at observed boundary 0.10 ng/mL | 0.794 (0.760-0.829) | <0.001 | 7,643 |
| Clinical pregnancy: Exclude records at observed boundary 0.10 ng/mL; primary conditional model | 0.689 (0.586-0.810) | <0.001 | 4,203 |
| Live birth: Exclude records at observed boundary 0.10 ng/mL; primary conditional model | 0.784 (0.666-0.924) | 0.004 | 4,203 |
| Recorded blastocyst yield: Replace 0.10 ng/mL with 0.010 ng/mL | 0.797 (0.764-0.832) | <0.001 | 7,778 |
| Clinical pregnancy: Replace 0.10 ng/mL with 0.010 ng/mL; primary conditional model | 0.694 (0.592-0.815) | <0.001 | 4,273 |
| Live birth: Replace 0.10 ng/mL with 0.010 ng/mL; primary conditional model | 0.796 (0.677-0.936) | 0.006 | 4,273 |
| Recorded blastocyst yield: Replace 0.10 ng/mL with 0.050 ng/mL | 0.797 (0.764-0.832) | <0.001 | 7,778 |
| Clinical pregnancy: Replace 0.10 ng/mL with 0.050 ng/mL; primary conditional model | 0.694 (0.592-0.815) | <0.001 | 4,273 |
| Live birth: Replace 0.10 ng/mL with 0.050 ng/mL; primary conditional model | 0.796 (0.677-0.936) | 0.006 | 4,273 |
| Recorded blastocyst yield: Replace 0.10 ng/mL with 0.099 ng/mL | 0.796 (0.763-0.831) | <0.001 | 7,778 |
| Clinical pregnancy: Replace 0.10 ng/mL with 0.099 ng/mL; primary conditional model | 0.694 (0.591-0.815) | <0.001 | 4,273 |
| Live birth: Replace 0.10 ng/mL with 0.099 ng/mL; primary conditional model | 0.796 (0.676-0.936) | 0.006 | 4,273 |

Denominator compatibility audit: the historical Day-3 proxy subtracted all recorded transferred embryos when Day_3=1. It retained 7,217 positive denominators and excluded 539 zero and 22 negative values. All 22 negative values had 2PN=1 and transfer count=2; the expanded source recorded no culture in these records, and 12 had thawing evidence. Twenty-three zero-proxy records had both positive culture and blastocyst counts; 224 retained records had blastocyst counts above the proxy. Original blastocysts exceeded 2PN in 173 records but were all compatible with recorded culture counts. Fertilization origins remain unlinked. These findings invalidate the subtraction assumption without establishing source-count error. Table S14 uses recorded culture count directly; neither proxy verifies 2PN-only biological blastulation.

**Table S8. Propensity-score common-support and balance summary.**

| **Diagnostic** | **Result** |
| --- | --- |
| Complete propensity-score cohort | 7,775 |
| Inside empirical common support | 7,754 (99.7%) |
| Outside empirical common support | 21 |
| Overlap-weighted effective sample size | 4607 |
| Maximum absolute weighted SMD, continuous covariates | 0.0014 |
| Maximum absolute weighted SMD, categorical levels | 0.0029 |

**Table S9. Internal bootstrap stability of the exploratory changepoint.**

| **Item** | **Result** |
| --- | --- |
| Bootstrap resamples | 1,000 |
| Valid estimates | 1,000 |
| Original R changepoint | 0.0711 |
| Reconstructed full-sample changepoint | 0.0687 |
| Bootstrap median | 0.0710 |
| Bootstrap interquartile range | 0.0630-0.0737 |
| Bootstrap 2.5th-97.5th percentiles | 0.0548-0.0857 |

**Table S10. Assumed temporal ordering and analytic classification of measured variables.**

| **Node class** | **Recorded variables** | **Role considered in analysis** | **Timing** | **Analytic handling** |
| --- | --- | --- | --- | --- |
| Baseline attributes | Age, BMI, infertility type/diagnosis, AMH, AFC, basal hormones, phenotype label, year | Candidate common causes | Pre-stimulation | Adjusted as measured; phenotype label not reconstructed |
| Stimulation process | Protocol, duration, gonadotropin dose | Treatment/process causes | Before trigger/retrieval | Included in primary and reduced models |
| Trigger-day state | Progesterone, E2, endometrial thickness | Exposure component/outcome predictors | Trigger day | P contributes to P/MII |
| Retrieval state | Oocytes retrieved, MII count | Exposure component and post-trigger state | After trigger | MII contributes to P/MII; causal status is mixed |
| Embryo development | 2PN, Day-3 embryos, blastocysts | Post-retrieval outcomes/mediators | After retrieval | Not baseline confounders |
| Recorded-transfer selection | Transfer >0; linked cancellation/deferral reasons | Selection/collider | After retrieval | Reasons summarized, not adjusted; no observed positive transfer in PPCOS-coded records |
| Transfer process | Transfer day, embryo number, good-quality embryo transfer | Post-exposure mediators/selection descriptors | At transfer | Included only in primary conditional model |
| Clinical outcomes | Implantation-positive cycle, clinical pregnancy, live birth | Outcomes | After transfer | Associational estimands only |

**Table S11. Transfer selection and data-integrity audit.**

| **Audit dimension** | **Level/finding** | **Positive transfer or count** | **Result/action** |
| --- | --- | --- | --- |
| P/MII group | ≤0.0711 | 3036/5408 | 56.1% |
| P/MII group | >0.0711 | 1237/2370 | 52.2% |
| Stimulation protocol | Agonist | 2821/4349 | 64.9% |
| Stimulation protocol | Antagonist | 1400/2672 | 52.4% |
| Stimulation protocol | Others | 52/230 | 22.6% |
| Stimulation protocol | PPCOS-coded | 0/527 | 0.0% |
| Phenotype label | NOR | 3181/5390 | 59.0% |
| Phenotype label | PCOS | 236/661 | 35.7% |
| Phenotype label | POR | 856/1727 | 49.6% |
| Indicator audit | Cancellation_rate=0 but transferred embryos=0 | 4 records | Reclassified as no transfer |
| Indicator audit | Cancellation_rate=1 but transferred embryos>0 | 4 records | Included in the positive-transfer record group |
| Transfer-day audit | Both Day-3 and Day-5 flags | 2 records | Classified Day 3 from cleavage-embryo type |

**Table S12. Audit of the recorded normal-fertilization-rate denominator.**

| **Pattern matching the recorded rate** | **Cycles** | **Percent** |
| --- | --- | --- |
| retrieved-oocyte-only match | 3,450 | 44.4% |
| both denominators match | 3,104 | 39.9% |
| MII-only match | 1,220 | 15.7% |
| neither denominator matches | 4 | 0.1% |
| Interpretation: the calculation pattern does not reliably identify IVF versus ICSI. Fertilization-method analyses therefore used the linked source-registry field, not a derived rate proxy. | | |

**Supplementary Figure S1. Conceptual causal structure for the calculated P/MII analyses.**

The diagram records the assumed ordering used to classify covariates and selection variables. P/MII is a deterministic child of trigger-day progesterone and post-retrieval MII count. The original diagram label Fresh-transfer selection describes the assumed treatment pathway; operationally, S=1 denotes a positive recorded transfer count and does not verify the fresh origin of every transferred embryo. Pregnancy models condition on this recorded transfer selection. Cancellation or transfer-deferral reasons describe that selection process and were not used as baseline adjustment variables. The diagram does not claim identification of a causal total effect. ICSI, intracytoplasmic sperm injection; IVF, in vitro fertilization; MII, metaphase II; OHSS, ovarian hyperstimulation syndrome; P, progesterone; PGT, preimplantation genetic testing; 2PN, two pronuclei. The diagram label post-retrieval P/MII refers to when the calculated ratio becomes available, not to a progesterone sample obtained after retrieval.

**Table S13. Source-registry linkage, fertilization-method sensitivity analyses, and cancellation or transfer-deferral reasons.**

**Panel A. Linkage and field completeness.**

| **Audit item** | **Result** |
| --- | --- |
| Unique source-registry ART cycles | 21,526 |
| Analytic cycles linked one-to-one | 7,778/7,778 |
| Fertilization method available | 7,778/7,778 |
| Transfer by positive transfer count | 4,273 |
| No recorded transfer | 3,505 |
| No-transfer cycles with recorded reason | 3,500 (99.9%) |
| Registry cancellation flag vs transfer-count disagreements | 8 |
| Uterine/cavity eligibility flags or sequential exclusion hierarchy | Not available |

**Panel B. Fertilization-method distribution by P/MII group.**

| **Method** | **P/MII** | **Cycles** | **Records with transfer** | **2PN** | **Blastocysts** |
| --- | --- | --- | --- | --- | --- |
| Conventional IVF | ≤0.0711 | 3,968 | 2,440 | 37,228 | 22,595 |
| Conventional IVF | >0.0711 | 1,482 | 914 | 6,108 | 2,438 |
| ICSI-based | ≤0.0711 | 1,439 | 596 | 13,208 | 6,820 |
| ICSI-based | >0.0711 | 888 | 323 | 3,743 | 1,489 |
| Mixed IVF+ICSI | ≤0.0711 | 1 | 0 | 22 | 18 |

**Panel C. Method-adjusted and method-stratified estimates for P/MII >0.0711 versus ≤0.0711.**

| **Outcome and stratum** | **Effect** | **Estimate (95% CI)** | **P** | **n** |
| --- | --- | --- | --- | --- |
| Recorded blastocyst yield: overall + fertilization method | aRR | 0.801 (0.767-0.836) | <0.001 | 7,778 |
| Clinical pregnancy: overall + fertilization method | aOR | 0.699 (0.595-0.821) | <0.001 | 4,273 |
| Live birth: overall + fertilization method | aOR | 0.798 (0.678-0.940) | 0.007 | 4,273 |
| Recorded blastocyst yield: Conventional IVF | aRR | 0.779 (0.738-0.821) | <0.001 | 5,450 |
| Clinical pregnancy: Conventional IVF | aOR | 0.691 (0.575-0.830) | <0.001 | 3,354 |
| Live birth: Conventional IVF | aOR | 0.775 (0.644-0.933) | 0.007 | 3,354 |
| Recorded blastocyst yield: ICSI-based | aRR | 0.845 (0.786-0.908) | <0.001 | 2,327 |
| Clinical pregnancy: ICSI-based | aOR | 0.763 (0.545-1.069) | 0.116 | 919 |
| Live birth: ICSI-based | aOR | 0.939 (0.664-1.326) | 0.719 | 919 |

**Panel D. Fertilization-method interaction tests.**

| **Outcome** | **Test** | **P interaction** | **n** |
| --- | --- | --- | --- |
| Recorded blastocyst yield | Robust Wald | 0.003 | 7,777 |
| Clinical pregnancy | Robust Wald | 0.972 | 4,273 |
| Live birth | Robust Wald | 0.935 | 4,273 |

**Panel E. Most frequent reasons among the 3,505 cycles without a recorded transfer.**

| **Reason** | **P/MII ≤0.0711** | **P/MII >0.0711** | **Total** |
| --- | --- | --- | --- |
| High risk of OHSS | 1,289 | 120 | 1,409 |
| PGT testing | 411 | 191 | 602 |
| Illness or other personal reasons | 191 | 207 | 398 |
| Hydrosalpinx | 195 | 123 | 318 |
| PGT pending | 60 | 147 | 207 |
| Stimulation under high progestogen levels (recorded reason) | 43 | 75 | 118 |
| Thin endometrium | 23 | 86 | 109 |
| Abnormal endometrial echogenicity | 41 | 21 | 62 |
| No transferable embryo | 6 | 31 | 37 |
| Adenomyosis pretreatment | 15 | 21 | 36 |
| Other or unrecorded reasons | 98 | 111 | 209 |

Reasons were obtained from the linked registry and are descriptive. They were not used as regression covariates because they arise after retrieval and form part of the transfer-selection mechanism. ICSI-based includes ICSI, rescue ICSI and late ICSI; the single mixed IVF+ICSI cycle was excluded from stratified and interaction analyses. Recorded no-transfer reason categories are distinct from stimulation-protocol categories and should not be interpreted as protocol counts.

Source scope: the original Table S13 panels use the 21,526-key registry. Tables S14-S16 use the newly linked 15,436-key clinical and laboratory export, which contains additional fields and all 7,778 analytic keys. The exports do not reconstruct the upstream selection sequence.

**Table S14. Recorded culture-count denominator sensitivities**

New post hoc Poisson models used the original embryological covariates and HC0 variance. Culture-positive denotes recorded extended-culture count >0; the comparable 2PN models use exactly the same 5,821 records. First-retrieval denotes recorded retrieval order 1; all 4,960 culture-positive records in that subset had no positive thaw count or valid thaw identifier. Counts and stored P/MII values were unchanged.

| **Population** | **Offset denominator** | **Contrast** | **N** | **aRR (95% CI)** | **P** |
| --- | --- | --- | --- | --- | --- |
| All culture >0 | Recorded culture count | High versus low | 5,821 | 0.974 (0.945-1.005) | 0.101 |
| All culture >0 | Recorded culture count | P/MII per 0.01 | 5,821 | 0.993 (0.989-0.997) | <0.001 |
| All culture >0 | Recorded 2PN | High versus low | 5,821 | 0.934 (0.902-0.968) | <0.001 |
| All culture >0 | Recorded 2PN | P/MII per 0.01 | 5,821 | 0.987 (0.982-0.991) | <0.001 |
| First retrieval; culture >0 | Recorded culture count | High versus low | 4,960 | 0.973 (0.940-1.006) | 0.108 |
| First retrieval; culture >0 | Recorded culture count | P/MII per 0.01 | 4,960 | 0.993 (0.989-0.997) | <0.001 |

High means P/MII >0.0711; low means ≤0.0711. Covariates: age, infertility duration, gonadotropin dose, stimulation protocol, infertility diagnosis, cycle year, retrieved-oocyte count, trigger-day E2, AFC and AMH. The full culture-positive population includes 132 thaw-positive records. The recorded culture count is internally compatible with blastocyst counts and the source available-blastocyst rate, but embryo-level pronuclear and retrieval origins were unavailable, limiting biological interpretation of the denominator. Selection into culture and incomplete embryo provenance remain. All six models converged without warnings.

**Table S15. Source-restricted transfer sensitivities**

New post hoc logistic models preserve the existing clinical-pregnancy and live-birth flags. Population A requires positive transfer, retrieval-to-transfer interval 3, 5 or 6 days, zero thaw count and no valid thaw identifier (n=4,258; lower/higher P/MII 3,033/1,225). Population B additionally requires recorded retrieval order 1 (n=3,526; lower/higher 2,657/869), with a distinct source identifier for every record. Date consistency and absent thaw evidence do not independently identify each embryo source.

| **Population** | **Recorded outcome** | **Contrast** | **N** | **aOR (95% CI)** | **P** |
| --- | --- | --- | --- | --- | --- |
| A: no recorded thaw | Clinical pregnancy | High versus low | 4,258 | 0.695 (0.592-0.816) | <0.001 |
| A: no recorded thaw | Clinical pregnancy | P/MII per 0.01 | 4,258 | 0.964 (0.946-0.983) | <0.001 |
| A: no recorded thaw | Clinical pregnancy | P/MII per 0.01 with P and MII | 4,258 | 0.973 (0.946-1.001) | 0.059 |
| A: no recorded thaw | Live birth | High versus low | 4,258 | 0.793 (0.674-0.934) | 0.005 |
| A: no recorded thaw | Live birth | P/MII per 0.01 | 4,258 | 0.978 (0.959-0.997) | 0.023 |
| A: no recorded thaw | Live birth | P/MII per 0.01 with P and MII | 4,258 | 0.990 (0.963-1.018) | 0.494 |
| B: first retrieval | Clinical pregnancy | High versus low | 3,526 | 0.633 (0.527-0.760) | <0.001 |
| B: first retrieval | Clinical pregnancy | P/MII per 0.01 | 3,526 | 0.951 (0.929-0.973) | <0.001 |
| B: first retrieval | Clinical pregnancy | P/MII per 0.01 with P and MII | 3,526 | 0.952 (0.919-0.986) | 0.006 |
| B: first retrieval | Live birth | High versus low | 3,526 | 0.740 (0.616-0.890) | 0.001 |
| B: first retrieval | Live birth | P/MII per 0.01 | 3,526 | 0.967 (0.944-0.989) | 0.004 |
| B: first retrieval | Live birth | P/MII per 0.01 with P and MII | 3,526 | 0.968 (0.935-1.002) | 0.067 |

Covariates: age, AFC, trigger-day E2, gonadotropin dose, stimulation protocol, retrieved-oocyte count, source-date transfer day, transferred-embryo count, and good-quality embryo transfer. Joint models additionally include P and MII. Variance is HC0. A has 2,363 clinical-pregnancy and 2,013 live-birth flags; B has 2,038 and 1,761, respectively. The displayed 12 models use source-date transfer day; an additional 12 models preserving the historical day derivation were also fitted and gave similar results. Only two source days differ from that derivation. All 24 models converged without warnings. A contains repeated source identifiers; B is unique on that field. Identifiers were treated as recorded grouping labels, with patient identity and clinical-event ascertainment addressed in the manuscript limitations.

**Table S16. Source-identifier grouping sensitivities**

**Panel A. Joint-component models with source-identifier-clustered covariance**

| **Recorded outcome** | **Records** | **Identifier groups** | **P/MII aOR per 0.01 (95% CI)** | **P** |
| --- | --- | --- | --- | --- |
| Clinical pregnancy | 4,273 | 3,912 | 0.971 (0.945-0.997) | 0.030 |
| Live birth | 4,273 | 3,912 | 0.988 (0.961-1.015) | 0.367 |

The historical primary-conditional formula and original transfer-day derivation were retained, with P/MII, P and MII jointly included. Cluster covariance used equality of the recorded source identifier, finite-group correction, and normal-reference confidence limits. Point estimates are unchanged from the original HC0 models. Source grouping is not independent confirmation of patient identity.

**Panel B. Fivefold source-identifier-grouped predictive performance**

| **Recorded outcome** | **Candidate model** | **Out-of-fold AUC** | **Out-of-fold Brier score** |
| --- | --- | --- | --- |
| Clinical pregnancy | Base | 0.6039 | 0.2376 |
| Clinical pregnancy | Base + P | 0.6097 | 0.2370 |
| Clinical pregnancy | Base + MII | 0.6109 | 0.2369 |
| Clinical pregnancy | Base + P + MII | 0.6157 | 0.2363 |
| Clinical pregnancy | Base + P/MII | 0.6170 | 0.2355 |
| Clinical pregnancy | Base + P + MII + P/MII | 0.6185 | 0.2353 |
| Live birth | Base | 0.6172 | 0.2383 |
| Live birth | Base + P | 0.6189 | 0.2382 |
| Live birth | Base + MII | 0.6220 | 0.2377 |
| Live birth | Base + P + MII | 0.6232 | 0.2376 |
| Live birth | Base + P/MII | 0.6228 | 0.2374 |
| Live birth | Base + P + MII + P/MII | 0.6243 | 0.2373 |

All candidates used the same outcome-specific StratifiedGroupKFold splits (five folds; shuffle=True; seed 20260815), keeping every source identifier in one fold. No identifier overlapped training and test sets. Base variables and within-fold scaling, one-hot encoding and logistic-regression settings match Table S6. These are new grouped sensitivities, separate from the original cycle-split outputs. The 4,273 records comprise 3,912 source identifiers. The identifier was used as a recorded grouping label; patient-level identity could not be independently established, as discussed in the manuscript limitations. Small AUC/Brier differences are descriptive; no paired uncertainty interval, superiority test, or external validation was performed. No fitting warnings were captured.
